# Supplementary material for: Novel Driver Strength Index highlights important cancer genes in TCGA PanCanAtlas patients
Source: PeerJ. 2022 Aug 11;10:e13860. doi: 10.7717/peerj.13860 (PMC9375969; doi:10.7717/peerj.13860)
Supplement: Supplemental Information 2 [file peerj-10-13860-s002.zip › COHORTS/genes plots/detailed/2021_8_16_14_9_distribution_events_detailed_31.pdf]

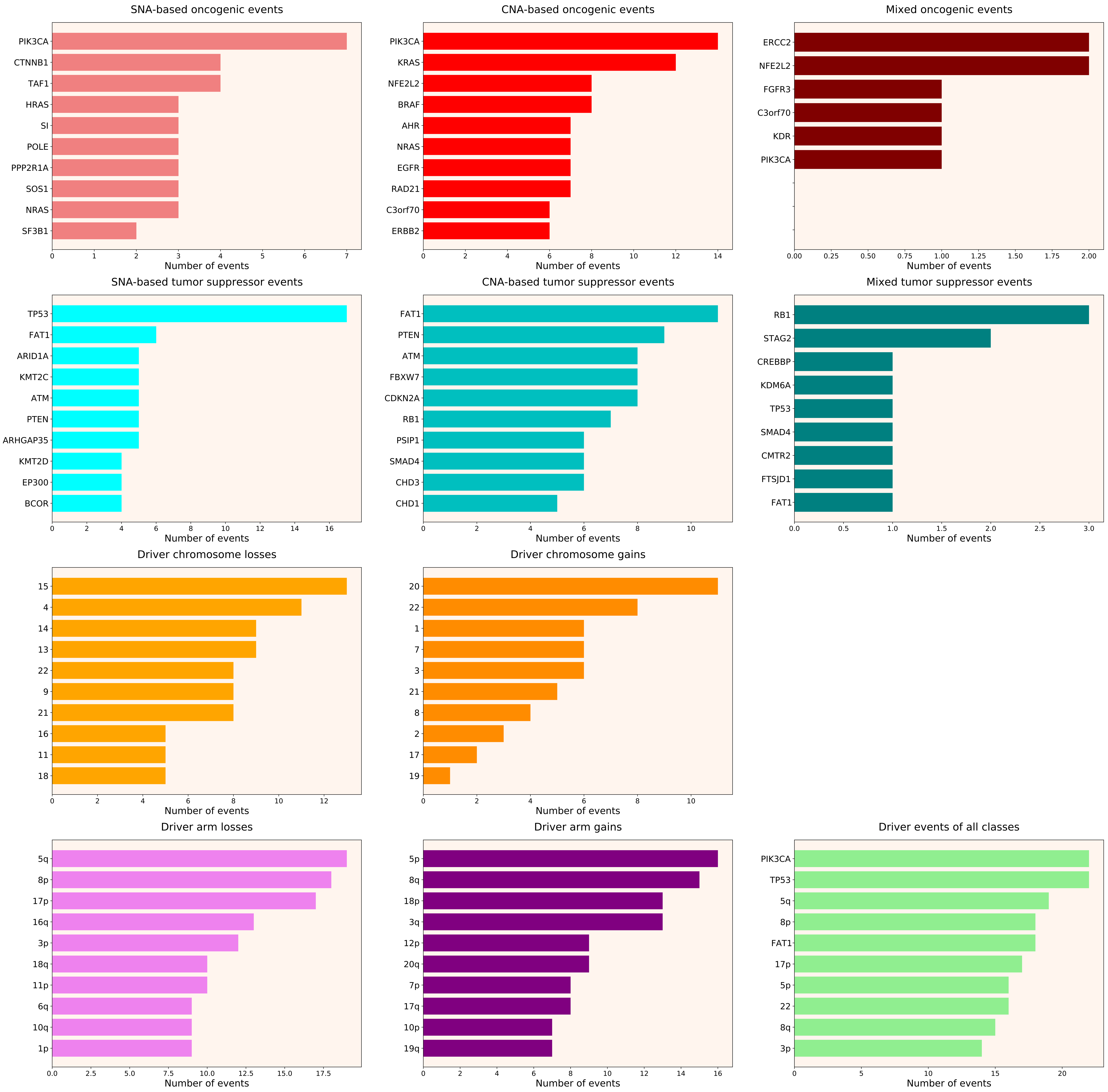
SNA-based oncogenic events
PIK3CA
CTNNB1
TAF1
HRAS
SI
POLE
PPP2R1A
SOS1
NRAS
SF3B1
Number of events
CNA-based oncogenic events
PIK3CA
KRAS
NFE2L2
BRAF
AHR
NRAS
EGFR
RAD21
C3orf70
ERBB2
Number of events
Mixed oncogenic events
ERCC2
NFE2L2
FGFR3
C3orf70
KDR
PIK3CA
Number of events
SNA-based tumor suppressor events
TP53
FAT1
ARID1A
KMT2C
ATM
PTEN
ARHGAP35
KMT2D
EP300
BCOR
Number of events
CNA-based tumor suppressor events
FAT1
PTEN
ATM
FBXW7
CDKN2A
RB1
PSIP1
SMAD4
CHD3
CHD1
Number of events
Mixed tumor suppressor events
RB1
STAG2
CREBBP
KDM6A
TP53
SMAD4
CMTR2
FTSJD1
FAT1
Number of events
Driver chromosome losses
15
4
14
13
22
9
21
16
11
18
Number of events
Driver chromosome gains
20
22
1
7
3
21
8
2
17
19
Number of events
Driver arm losses
5q
8p
17p
16q
3p
18q
11p
6q
10q
1p
Number of events
Driver arm gains
5p
8q
18p
3q
12p
20q
7p
17q
10p
19q
Number of events
Driver events of all classes
PIK3CA
TP53
5q
8p
FAT1
17p
5p
22
8q
3p
Number of events
